# A decade of the ORCHESTRA study: organizational characteristics, patient outcomes, performance and efficiency in critical care

**DOI:** 10.62675/2965-2774.20240118-en

**Published:** 2024-07-01

**Authors:** Marcio Soares, Jorge Ibrain Figueira Salluh, Fernando Godinho Zampieri, Fernando Augusto Bozza, Pedro Martins Pereira Kurtz

**Affiliations:** 1 Instituto D’Or de Pesquisa e Ensino Rio de Janeiro RJ Brazil Instituto D’Or de Pesquisa e Ensino - Rio de Janeiro (RJ), Brazil.; 2 Faculty of Medicine and Dentistry University of Alberta Edmonton Canada Faculty of Medicine and Dentistry, University of Alberta - Edmonton, Canada.

## INTRODUCTION

The organization and structure of intensive care units (ICUs) affect the quality and efficiency of critical care.^(1,2)^ Because acute care delivery varies significantly across countries, patient populations and local care practices, the associations of ICU structure, process and outcomes are also expected to differ depending on the context. Currently, most of the available information on ICUs has been reported in studies performed in developed countries, and these results may not fully translate to developing countries.

### A brief history of the ORCHESTRA Study

**Figure 1:**
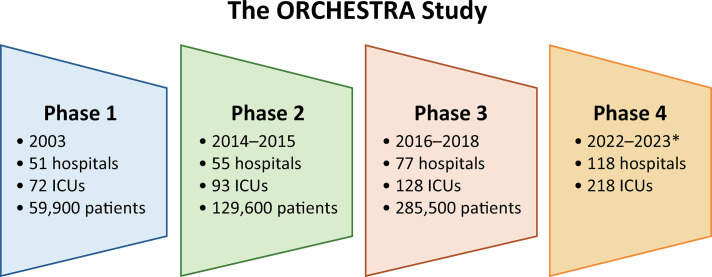
Number of hospitals, intensive care units and patients included in each of the four ORCHESTRA phases. The study was not performed between 2019 and 2021. * Phase 4 is still ongoing. The final number of hospitals, intensive care units and patients is not yet known. ICU - intensive care unit.

To help bridge the above mentioned gap, in 2014, the ORCHESTRA (ORganizational CHaractErSTics in cRitical cAre) study was designed to describe the structure, process and organization of ICUs and to investigate the impact of these characteristics on patient outcomes and on performance and efficiency of critical care. The study was planned in phases to propose hypotheses consistent with current knowledge and to include new centers and patients in each phase. At the beginning, the study included exclusively Brazilian ICUs, but in more recent phases, ICUs from Uruguay were also included. The number of patients and centers included in all phases is shown in figure 1. In the first three phases, more than 475,000 patients were included across more than 200 ICUs. Nonetheless, the study paused during the coronavirus disease 2019 (COVID-19) pandemic because ICU and hospital organizations were severely affected; furthermore, the patients’ case data were also affected.^(3)^The fourth phase is currently ongoing and includes ICU admissions from 2022 to 2023. The list of centers and investigators participating in all phases is provided in Appendix 1S (Supplementary Material).

### Study design and methodology

A full description of the methods is provided in Appendix 2S (Supplementary Material). Briefly, the ORCHESTRA study is a multicenter retrospective cohort study that used prospectively collected data from consecutive ICU admissions. The study uses a pragmatic approach. ICUs registered in the Brazilian Research in Critical Care Network (BRICNet)^(4)^ that use the Epimed Monitor System (Epimed Solutions, Rio de Janeiro, Brazil),^(5)^ a commercial cloud-based registry for quality improvement and benchmarking purposes, are invited to participate. The deidentified data of all adult (≥ 16 years old) patients are retrieved from the Epimed Monitor System®. Patients who were readmitted or whose core data (e.g., admission diagnosis and hospital outcomes) were missing are excluded. Data are prospectively entered in a structured electronic case report form by a combination of data integrated with local electronic health records and data entered manually by a trained case manager. The collected data include demographics, diagnoses, comorbidities and frailty assessments; scores used regularly in critical care, including the Simplified Acute Physiology Score (SAPS) 3; use of organ support; and ICU and hospital outcomes, among other variables. All variables in the Epimed Monitor System are structured with internal linked codes with no free text fields, with procedures and controls to assist data entry and minimize processing errors and the recording of outlying or implausible values.

Subsequently, the ICU director and/or chief nurse complete an online survey about hospital and ICU organizational, structural and process characteristics. The domains of the survey are based on literature in all study phases and include, for instance, ICU and hospital characterizations, staffing patterns, multidisciplinary rounds, use of checklists, implementation of protocols to prevent health care-associated complications, and family care policies. The primary outcome of the study is in-hospital mortality. The secondary outcomes include ICU mortality, ICU stay and hospital length of stay. In addition, measures of ICU performance and efficiency are evaluated.^(6,7)^

Patients’ and ICUs’ deidentified data are centrally processed and analyzed in dedicated servers with control of accesses and logs in compliance with data privacy and protection regulations.

### Main publications and results

Table 1S of the Supplementary Material summarizes the results of all ORCHESTRA-related publications; however, there are some ongoing studies. Here, we present a selected sample of the main findings.

In the first study, the number of fully implemented protocols and jointly (more than one care provider involved) managed clinical protocols were associated with lower mortality.^(8)^ In a subsequent study in patients with cancer, in addition to the number of protocols, the presence of dedicated pharmacists in the ICU and the occurrence of daily meetings between oncologists and intensivists for care planning were associated with lower mortality rates and more efficient resource use.^(9)^ Checklists and protocols are also essential for guaranteeing the continuity of quality of care during weekends, particularly for scheduled surgical patients.^(10)^ The implementation of protocols associated with better outcomes is a recurring finding of several ORCHESTRA-related studies (Table 1S - Supplementary Material) and contrasts with the findings from studies carried out in developed countries.^(11,12)^ We can hypothesize that in a scenario with a lower intensity of nurses and other care providers per patient, protocolized processes to prevent health care-associated complications and to adhere to best-evidence practices contribute to mitigating the effects on the continuity of care. Cluster analysis using machine learning was used to investigate whether staffing-related patterns were associated with improved outcomes.^(13)^ Intensive care units belonging to the cluster with full-time intensivists, dedicated pharmacists and higher levels of nurse autonomy had the best outcomes.

We also took the opportunity to evaluate and validate several scores used routinely in critical care. For instance, the SAPS 3, recommended by the *Associação de Medicina Intensiva Brasileira* (AMIB) to evaluate ICU performance in Brazil, was only validated in studies with a limited number of institutions and patients. A validation study using the ORCHESTRA database revealed that the SAPS 3 standard equation was well adjusted for recommendation in Brazil. Notably, we also validated the standardized resource use ratio as a measure of efficiency in resource use in the ICU.^(8,14)^

## CONCLUSION

The ORCHESTRA study is one of the largest contemporary cohort studies worldwide and has contributed to identifying potentially modifiable targets to improve ICU organization and patient care. Furthermore, it has been interesting to validate relevant instruments to characterize and stratify critically ill patients and to assess ICU performance and efficiency. Future perspectives for the next phases include ICUs from other countries where the study eligibility criteria can be met.
