# Supplementary material for: A decade of the ORCHESTRA study: organizational characteristics, patient outcomes, performance and efficiency in critical care
Source: Crit Care Sci. 2024 Jul 1;36:e20240118en. doi: 10.62675/2965-2774.20240118-en (PMC11239203; doi:10.62675/2965-2774.20240118-en)
Supplement: Supplementary file 1 [file 2965-2774-ccsci-36-e20240118en-suppl01.pdf]

Critical Care Science
https://doi.org/10.62675/2965-2774.20240118-en

SUPPLEMENTARY MATERIAL

A decade of the ORCHESTRA study: organizational characteristics, patient outcomes, performance and efficiency in critical care

Marcio Soares[1], Jorge Ibrain Figueira Salluh[1], Fernando Godinho Zampieri[2], Fernando Augusto Bozza[1], Pedro Martins Pereira Kurtz[1], on behalf of the ORCHESTRA Study Investigators

APPENDIX 1S - LIST OF CENTERS AND INVESTIGATORS IN EACH STUDY PHASE

The ORCHESTRA - Phase 1 (2013)

Steering Committee: Marcio Soares (principal investigator), Jorge Ibrain Figueira Salluh, Fernando Augusto Bozza (*Instituto D'Or de Pesquisa e Ensino*, Rio de Janeiro); Derek C. Angus, Jeremy M. Kahn (CHRISMA, University of Pittsburgh Medical Center, Pittsburgh)

Data Management and Secretariat: Grazielle V. Ramos, Aline R. Silva (*Instituto D'Or de Pesquisa e Ensino*, Rio de Janeiro)

Statistical analyses: Marcio Soares, Pedro E. A. A Brasil *(Instituto D'Or de Pesquisa e Ensino*, Rio de Janeiro)

Investigators and participating centers

Bahia - *Hospital Agenor Paiva*: Maristela Medeiros Machado, Josianne Souza Guimarães, Maria Beatriz Fauaze; **Ceará** - *Hospital Regional do Cariri*: Meton Soares de Alencar, Ivo Saturno Bonfim, Francisco Luciano F. F. de Souza; **Distrito Federal:** *Hospital Anchieta*: Rubens Antônio Bento Ribeiro, Rodrigo dos Santos Biondi, Raquel Fonseca, Fábio Ferreira Amorim, Eduardo Cesar Guimarães Lessa, Auriane Teixeira Barcelar; *Hospital do Coração do Brasil - Brasília*: Hélia Beatriz Nunes de Araújo, Leandro Mações Goulart, Yara Santos Aguiar; *Hospital Santa Luzia*, Brasília: Marcelo de Oliveira Maia, Gisele Brocco Maganan; **Espírito Santo** - *Hospital Unimed Vitória*: Eliana Bernadete Caser, Silvane Damasceno; **Maranhão** - *Hospital Geral Tarquínio Lopes Filho*: Ana Paula Pierre de Moraes; *UDI Hospital*: Alexandre Guilherme Ribeiro de Carvalho, Ana Cláudia Pinho de Carvalho, Edilene Coelho de Souza Novaes; **Minas Gerais** - *Santa Casa de Caridade de Diamantina*: Marcelo Ferreira Sousa, José Aristeu de Andrade, Marcelo Ferreira Sousa, Ana Luiza Dayrell Gomes da Costa Sousa, Marcia Maria Ferreira de Souza; *Santa Casa de Misericórdia de Juiz de Fora*: Guilherme Côrtes Fernandes, Wilson Coelho Pereira Neto, Guillermo Patricio Ortega Jacome, Meire Cavalieri de Almeida, Maria do Socorro Van Keulen; **Paraíba** - *Hospital Universitário Lauro Wanderley*: Ciro Leite Mendes, Paulo César Gottardo; **Pernambuco** - *Hospital Esperança*: Mariza da Fonte Andrade Lima, Marçal Paiva, Cristiane Mendes, Maria de Fátima Mesquita, Roberto de Oliveira Burel; *Hospital Prontolinda*: Michele Godoy, Gustavo Rocha, Tatiana Cadena, Leonardo Diamante, Lanecley Fulco, Michele Godoy, Jeanine Guerra; *Hospital São Marcos*: Sérgio Holanda Cavalcanti, Luciane Ishy, João Rodolfo, Weidson Dantas, Bárbara Souza; **Rio de Janeiro** - *Hospital Bangu*: Sandro Vieira de Oliveira, Adriana Campos; *Hospital Barra D'Or*: Walter Homena Jr, Francisco Gabriel, Marcelo Santino, Diamantino Salgado, Carlos André; *Hospital Copa D'Or*: William Nascimento Viana, Cecilia Rosa Magno, Marco Aurélio Fernandes, *Álvaro Pontes*, Maria Eduarda Tavares, Felipe Saddy, William Nascimento Viana, Enio Gustavo Schroedder; *Hospital de Clínicas Mario Lioni*: Marcelo Elysio Lugarinho; *Hospital de Clínicas de Niterói*: Moyzes Damasceno, Felipe Ribeiro, Diogo Terrana, Valdênia Pereira de Souza, Angelo Michele di Candia; *Hospital do Câncer II, Instituto Nacional de Câncer*: Rodolfo Spinoza, José Jorge Netto; *Hospital Israelita Albert Sabin*: Alexandre Vaz Scotti; *Hospital Municipal Souza Aguiar*: Roberto Seabra Lannes, Sion Divan Filho, Andrea Ludovico; *Hospital Norte D'Or*: Jorge Eduardo da Silva Soares Pinto; *Hospital Oeste D'Or*: Márcia Adélia de Magalhães Menezes, Rosa Stancato, Carolina Saturnino Braga, Joyce Andrade, Guilherme Brenande Alves Faria, Liliane R. de Mendonça, Alcino

This is an open access article under the CC BY license (https://creativecommons.org/licenses/by/4.0/).

Márcio Toledo; *Hospital Pasteur*, Rio de Janeiro: Pedro Alberto Varaschin, Renata Orofino, Anna Luida Mundt; *Hospital Quinta D'Or*: Roberto Costa, Cristiane Belo, Eduardo Augusto de Oliveira Xavier, *Márcio Niemeyer Guimarães*, Cristiane Carius de Oliveira, Juliana G. da Silveira, Leonardo Campioni, Alessandra Gouvêa Longo; *Hospital Rios D'Or*: Alessandra Alves; *Hospital Samaritano*: Ricardo Lima; *Hospital São Lucas Copacabana*: Marcos Knibel, Pedro Azambuja, Christian Nejm Roderjan; *Hospital Total Cor*: Rodrigo Hatum, André Japiassú; *Clínica São Vicente*: Patrícia Soares D'Alessandro, Arthur Vianna; *Centro Hospitalar do Instituto de Pesquisa Clínica Evandro Chagas*, *Instituto Nacional de Infectologia*, *Fiocruz*: Denise Machado Medeiros, André Miguel Japiassú; **Rio Grande do Sul** - *Santa Casa de Porto Alegre - Hospital Dom Vicente Scherer*: Thiago Lisboa, Edison Moraes Rodrigues Filho; *Santa Casa de Porto Alegre - Hospital Santa Rita*: Thiago Lisboa, André Peretti Torelly; *Santa Casa de Porto Alegre - Pavilhão Central*: Thiago Lisboa, Jorge Amilton Hoher; *Santa Casa de Porto Alegre - Pavilhão Pereira Filho*: Thiago Lisboa, Paula Pinheiro Berto, Jéssica Oliveira; **São Paulo** - *Hospital de Câncer de Barretos, Fundação Pio XII*: Ulysses Vasconcellos de Andrade e Silva; *Hospital Alemão Oswaldo Cruz*: Fernando Colombari; HCor-*Hospital do Coração*: Alexandre Biasi Cavalcanti, Edson Renato Romano, Rosianne Vasconcelos; *Hospital de Base de São José do Rio Preto*: Suzana M. A. Lobo; *Hospital Israelita Albert Einstein*: Thiago Domingos Corrêa, Eliezer Silva; *Hospital São Camilo Pompeia*: Alexandre Toledo Maciel; *Hospital São Francisco*: Marcus Antônio Ferez, Edson Antonio Nicolini, Brunno de Oliveira Silva, Kamila da Grazia Iazzetta; *Hospital São Luiz - Unidade Anália Franco*: André Luiz Baptiston Nunes, Rafaela Deczka Morsch; *Hospital São Luiz - Unidade Brasil*: Haggeas da Silveira Fernandes; *Hospital São Luiz - Unidade Itaim*: Leonardo Brauer; *Hospital São Luiz - Unidade Jabaquara*: Bruno Franco Mazza, *Débora Dutra da Silveira Mazza*, Roberto Álvaro Ramos Filho; *Hospital São Luiz - Unidade Morumbi*: Bruno Franco Mazza, *Débora Dutra da Silveira Mazza*; *Hospital Sírio-Libanês*: Luciano Cesar Pontes Azevedo, Guilherme Schettino; *Hospital Vivalle*: Fernando Vinicius Cesar de Marco, Guilherme Paro de Toledo Barros

The ORCHESTRA - Phase 2 (2014-2015)

Steering Committee: Marcio Soares (principal investigator), Jorge Ibrain Figueira Salluh, Fernando Augusto Bozza (*Instituto D'Or de Pesquisa e Ensino*, Rio de Janeiro); Fernando Godinho Zampieri (*HCor-Hospital do Coração*, São Paulo)

Data Management and Secretariat: Grazielle V. Ramos, Aline R. Silva (*Instituto D'Or de Pesquisa e Ensino*, Rio de Janeiro)

Statistical analyses: Marcio Soares, Fernando Godinho Zampieri, Otávio T. Ranzani

Investigators and participating centers

Bahia - *Hospital Agenor Paiva*: Maristela Medeiros Machado; *Hospital Santa Helena*: Luciano Ferreira de Souza, Maristela Medeiros Machado; **Distrito Federal** - *Hospital Anchieta*: Rubens Antônio Bento Ribeiro, Eduardo Cesar Guimarães Lessa; *Hospital Brasília*: Clayton Barbieri de Carvalho, Tullio Xavier Leirias; *Hospital Santa Luzia*, *Rede D'Or São Luiz DF*: Marcelo de Oliveira Maia, Edmilson Leal Bastos, Rebeca Martins da Silva Barros, Cintya M. V. Oliveira, Jose Aires A Neto; **Espírito Santo** - *Hospital Unimed Vitória*: Eliana Bernadete Caser, Silvane Damasceno; **Goiás** - *Hospital Geral de Goiânia*: Marcelo Rabahi Fouad, Marco Antônio Mendes Castilho, Durval Ferreira Fonseca Pedroso; Humberto Borges Barbosa; **Maranhão** - *Hospital de Câncer do Maranhão Tarquínio Lopes Filho*: Ana Paula Pierre de Moraes; *UDI Hospital*: Ana Cláudia Pinho de Carvalho, Alexandre Guilherme Ribeiro de Carvalho, Akemy Carvalho do Rosário; **Minas Gerais** - *Santa Casa de Caridade de Diamantina*: Marcelo Ferreira Sousa, Marcia Maria Ferreira de Souza; *Hospital das Clínicas da Universidade Federal de Minas Gerais*: Saulo Fernandes Saturnino; **Paraíba** - *Hospital Universitário Lauro Wanderley*: Ciro Leite Mendes, Paulo César Gottardo, Igor Mendonça do Nascimento; **Pernambuco** - *Hospital Esperança*: Mariza da Fonte Andrade Lima, Marçal Paiva; *Hospital Esperança Olinda*: Carlos Eduardo Ferraz Freitas, Lanecley Gouveia Neves Fulco; *Hospital São Marcos*: Maurício Magalhães Cabral, Luciane Ishiy, Renato Fábio Alberto Della Santa Neto; **Rio de Janeiro** - *Hospital Estadual Getúlio Vargas*: Giulliana Martines Moralez, Flavio Callil, Claudio Eduardo Calife Chagas, Eliane Casanova, Antonio Carlos Babo Rodrigues, Bruno Vidal; *Clínica São Vicente*: Arthur O. A. Vianna, Patrícia Soares D'Alessandro; *Hospital Estadual Adão Pereira Nunes*: Robson Correa Santos, Ricardo Pessoa Martelo; *Hospital Estadual Carlos Chagas*: Rodrigo Barros, Luisa Chuairi; *Hospital Quinta D'Or*: Roberto Costa, Cristiane Belo, Giulia P. C. Lima, Cristiane Cariús, Eduardo Xavier, Claudia Lourenço de Almeida, Rafael Sibanto, Alessandra Longo, Joyce Roma, Juliana Gurgel da Silveira, Laura Brasil Herranz, Gustavo Vaz, Bruno Cartelo Branco, Leonardo

Campioni, Alexandre Coscia; *Hospital Barra D'Or*: Walter Homena, Marcelo de Sousa Santino, Juan Carlos Verdeal; *Hospital Copa D'Or*: William Nascimento Viana, *Lígia* Sarmet Farah Cunha Rabello, Janaína Oliveira, Cecília Magno, Alex Gaspar, Guilherme Feres, Maria Teresa Saint-Martin; *Hospital Caxias D'Or*: Eric Perecmanis; *Hospital Norte D'Or*: Jorge Eduardo da Silva Soares Pinto, Sergio Teixeira Sant'Anna Junior; *Hospital Oeste D'Or*: Guilherme Brenande Alves Faria, Alcino Márcio Toledo de Medeiros, *Márcia Adélia de Magalhães Menezes, Rosa Imaculada* Stancato, Joyce Andrade; *Hospital Rios D'Or*: Alessandra Alves; *Hospital Badim*: Alexandre Vaz Scotti; *Hospital Municipal Souza Aguiar*: Roberto Seabra Lannes, Sion Divan Filho, Andrea Ludovico; *Hospital São Lucas*: Marcos Knibel, Emir Oliveira, Pedro Azambuja, Aline Affonso; *Hospital Unimed Costa do Sol*: Joel Tavares Passos; *Hospital Niterói D'Or*: Carlos Cesar Hortala Junior; *Hospital Israelita Albert Sabin*: Edmundo de Oliveira Tommasi, Patricia Frascari Litrento, Alexandra Gonçalves da Silva; *SAMER Hospital*: Henrique Miller Balieiro, Fellipe de Freitas Pereira; *Hospital Estadual Alberto Torres*: Ulisses de Oliveira Melo, Edson Tristão, Kelsey Sampaio, Rogerio Silveira, Antonio Carlos, Felipe Mafort, Jose Hipólito, Valquíria Queiroz; *Instituto Nacional de Câncer - HC II*: Bruno Azevedo da Cruz, Karla Biancha Silva de Andrade; **Rio Grande do Sul** - *Hospital Santa Rita*, *Santa Casa de Misericórdia de Porto Alegre*: Thiago Lisboa, André P. Torelly; *Santa Casa de Misericórdia de Porto Alegre, Pavilhão Pereira Filho*: Daniella Birriel; *Hospital Dom Vicente Scherer*, Santa Casa de Misericórdia de Porto Alegre: Edison Moraes Rodrigues Filho; *Hospital Montenegro*: José Pettine, Moreno Calcagnotto dos Santos, Tiago Almeida Ramos, Fernando Bourscheit, Ana Flávia Gallas Leivas; **São Paulo** - *Hospital São Francisco*: Marcus Antonio Ferez, Edson Antonio Nicolini; *Hospital Vivalle*: Fernando Vinicius Cesar de Marco, Guilherme Paro de Toledo; *Hospital de Câncer de Barretos, Fundação Pio XII*: Ulysses V. Andrade e Silva, Cristina Prata Amendola; *Hospital Alemão Oswaldo Cruz*: Fernando Colombari; *Hospital Israelita Albert Einstein*: Thiago Domingos Corrêa, Eliézer Silva; *Hospital Sírio-Libanês*: José Mauro Vieira Jr, Luciano Azevedo, Fernando Ramos; *Hospital São Luiz - Unidade Assunção*: Silvia Regina Ramos, Lilian Mara Perroud Miilher; *Hospital Sepaco*: Flávio Geraldo Rezende de Freitas, Antônio Tonete Bafi, Eduardo Souza Pacheco; *Hospital Santa Paula*: Dieter Eduardo Siefeld Araya, Ronaldo Escudeiro Borba, Moacyr Fogolin Junior, Pedro Ivo Buainain, Mariza Luciana Pregun; *Hospital do Rim*: Flávio Geraldo Rezende de Freitas, Antônio Tonete Bafi; *Rede D'Or São Luiz - Unidade Morumbi*: José Albani Carvalho Jr; *Rede D'Or São Luiz - Unidade Itaim*: José Albani Carvalho Jr, Mariza Silva Ramos Loesch, Kassia Pinho; *Hospital Samaritano*: Bruno Franco Mazza, Samantha Longhi de Almeida, Rosa Goldstein Alheira Rocha; *HCor-Hospital do Coração*: Edson Romano, Fernando Zampieri; *Hospital Nove de Julho*: Carlos Eduardo Nassif Moreira; *Hospital da Luz - Vila Mariana*: Bruno Adler Maccagnan Pinheiro Besen, Carlos Eduardo Brandão

The ORCHESTRA - Phase 3 (2016-2018)

Steering Committee: Marcio Soares, Fernando Augusto Bozza, Jorge Ibrain Figueira Salluh, Pedro Martins Pereira Kurtz, Gastón Burghi

Research Coordinators: Aline Reis da Silva Antunes, Grazielle Viana Ramos

Statisticians: Leonardo dos Santos Lourenco Bastos, Lunna Perdigão Borges, Fernando Godinho Zampieri

Investigators and participating centers

Brazil

Alagoas - *Hospital Memorial Arthur Ramos*: Maristela Medeiros Machado, Luciano Ferreira de Souza, Diogo Brandão, Maria Julia, Thiago Fortes, Milena Barbosa; **Bahia** - *Hospital Agenor Paiva*: Maristela Medeiros Machado, Marcos Souza, Galeno Magalhães Neto, Alexandre Boasorte, Josianne Guimarães, Sara Gonçalves; *Hospital Santa Helena*: Luciano Ferreira de Souza, Maristela Medeiros Machado, Maria Virgínia Barreto, João Claudio Lyra; **Distrito Federal** - *Hospital Brasília:* Clayton Barbieri de Carvalho; *Hospital Santa Luzia*: Marcelo de Oliveira Maia, Cyntia Mendes Vieira e Oliveira, Carlos Darwin G Silveira, Fabio F. Amorim, Jose Aires A. Neto; **Espírito Santo** - *Hospital Unimed Vitória*: Eliana Bernadete Caser, Silvane Damasceno; Wylcker Gustavo Wagnaker; **Goiás** - *Hospital Geral de Goiânia*: Marcelo Fouad Rabahi, Durval Ferreira F. Pedroso, Marco Antônio Castilho, Eros de Sousa Junior; **Maranhão** - *Hospital de Câncer do Maranhão Tarquínio Lopes Filho*: Ana Paula Pierre de Moraes, Gustavo Teixeira Alves; *Hospital São Domingos*: José Raimundo Araújo de Azevedo, Luís Eduardo França Tupinambá, Monique Silva Rocha , Hugo Leonardo de Jesus Gama , Hugo César Martins Lima, Danielle Cardoso de Macedo; *UDI Hospital*: Alexandre Guilherme Ribeiro de Carvalho, Brubo Ferreira de Carvalho; **Minas Gerais** - *Hospital das Clínicas da Universidade Federal de*

Minas Gerais: Saulo Fernandes Saturnino; *Hospital Felício Rocho*: Thaís de Paula Guimarães, Rogério de Castro Pereira, Sinval Lins da Silva, Daniel Fontes, Sarah de Campos Vicente, Rodrigo Silveira Santos; *Hospital Lifecenter*: Saulo Fernandes Saturnino; *Santa Casa de Caridade de Diamantina*: Marcelo Ferreira Sousa, Fabiana Souza Máximo Pereira, Marcia Maria Ferreira Souza, Fernanda Ribas Bernardes, Raquel Aparecida Monteiro, Renan Santiago Faria; **Pará** - *Hospital Adventista de Belém*: Edgar de Brito Sobrinho, Adriana de Oliveira Lameira Veríssimo, Monique Freitas de Albuquerque Ferreira; **Paraíba** - *Hospital Nossa Senhora das Neves*: Paulo César Gottardo, Ciro Leite Mendes, Elbia Assis Wanderley, Igor Mendonça do Nascimento, Katyucia Egito de Araújo Urquiza; *Hospital Universitário Lauro Wanderley*: Ciro Leite Mendes, Paulo César Gottardo, Igor Mendonça do Nascimento; **Pernambuco** - *Hospital Esperança,* Recife: Mariza da Fonte de Andrade Lima, Marçal Paiva Junior, Heloisa Ramos de Lacerda; *Hospital Santa Joana Recife*: Gustavo Antônio da Trindade Meira Henriques-Filho, Odin Barbosa da Silva, Rossana Saboya Leitão, Marcos Antônio Cavalcanti Gallindo, Arthur Henrique do Valle de Faria, Rafaella Milet Ferreira, Hermilo Borba Griz, Carlos Frederico Costa Lopes, Ana Luiza Medeiros Vasconcelos Lima, Danielle Ferraz Oliveira Aguiar; *Real Hospital Português de Beneficência Portuguesa*: Genes Felipe Rocha Cavalcanti, Noel Guedes Loureiro; **Rio de Janeiro** - *Complexo Hospitalar Américas, Hospital Samaritano*: Victor Cravo, Emmanuel Salgueiro; *Complexo Hospitalar Américas, Hospital Vitória*: Victor Cravo, Emmanuel Salgueiro; *Hospital Badim*: Alexandre Vaz Scotti, Antonino Galdino, Maria Inês Bissoli, Fábio Guilherme Santoro; *Hospital Barra D'Or*: Marcelo de Sousa Santino, Juan Carlos Rosso Verdeal, Gloria Adriana Rocha Martins, Walter Homena Jr, Marcelo Felix, José Alexandre Espósito, Francisco Gabriel, Paula Gorgulho, *Hospital Copa D'Or*: William Nascimento Viana; *Hospital Copa Star*: Pedro Kurtz, Fabio Miranda, Luiz Fernando Simvoulidis; *Hospital da Praia Brava:* Viviane Bogado Leite Torres, Davi Christ Fassano Cesar; *Hospital e Maternidade Santa Lúcia*: Celina Acra, Rodrigo Amâncio, Giulliana Martinez Moralez, Bruna Rajão Costa Fernandes, Tatiana Salgueiro, Maria Fernanda Camargo Barreto; *Hospital Estadual Alberto Torres:* Ulisses de Oliveira Melo; *Hospital Israelita Albert Sabin*: Edmundo de Oliveira Tommasi; *Hospital Mario Lioni*: Vinicius de Moraes, Edgard Carmo Neto, Fernando Pinto, Luiza Lourenço Lavoura Dias; *Hospital Municipal Souza Aguiar:* Roberto Seabra Lannes, Sio Divan Filho, Andrea Ludovico; *Hospital Norte D'Or*: Jorge Eduardo da Silva Soares Pinto, Sergio Teixeira Sant'Anna Junior; *Hospital Oeste D'Or*: Márcia Adélia de Magalhães Menezes, Guilherme Brenande Alves Faria, Joyce Andrade, Rosa Stancato, Carlos Henrique Ferreira Ramos, Alcino Marcio Toledo de Medeiros; Reinaldo Campos Rodrigues, Liliane Rodrigues de Mendonça; *Hospital Pasteur*: Pedro Alberto Varaschin, Cristiane Castanho, Pedro Henrique Barbosa de Almeida, Julia Barros Vargas, Fernando Alves Rocha, Diogo Salles; *Hospital Pró Cardíaco*: Rubens Carmo Costa Filho, Flavio Nácul, Carla Faria, Sonia Simões, Marcela Calomeni, Felipe Saddy, Fabio Reis; *Hospital Quinta D'Or*: Roberto Costa, Cristiane Belo, Cristiane Cariús, Claudia Lourenço de Almeida, Alessandra Longo, Juliana Gurgel da Silveira, Gustavo Vaz, Leonardo Campioni; *Hospital Rios D'Or*: Alessandra Alves; *Hospital São Lucas Copacabana*: Aline Affonso Carvalho, Marcos Knibel, Christian Nejm Roderjan; *Hospital Unimed Costa do Sol*: Joel Tavares Passos; *SAMER Hospital*: Henrique Miller Balieiro, Marcela Thevenet, Julia Meireles, Renata Junqueira; **Rio Grande do Norte** - *Hospital Promater*: Eduardo Queiroz da Cunha, Laís Mayara da Silva; **Rio Grande do Sul** - *Hospital de Clínicas de Porto Alegre*: Márcio Manozzo Boniatti, Thiago Costa Lisboa, Cristiano Augusto Franke, Edison Moraes Rodrigues Filho, Fabiano Marcio Nagel; *Hospital Mãe de Deus*: Helen Martins Valentim, Eduarda Cristina Martins, Lilian Silveira; *Hospital São Lucas da PUCRS*: Cassiano Teixeira, Renata Farion do Nascimento, Luciana Maria Caccavo Miguel, Luiz Gustavo Marin, Mariana Nunes Ferreira Jost, Raquel Kohman de Medeiros, Julia Primo, Martha Primo, Janaina Elsing, Ana Luiza Rizzatti Filipini, Gabriele Lobato Marins, Danielle Molardi de Aguiar, Luciano Marini; *Santa Casa de Porto Alegre - Hospital Santa Rita*: Thiago Lisboa, André Torelly, Martha Hadrich, Caroline Fachini; **São Paulo** - Hospital Alvorada Moema: Alexandre Habitante, Mario Henrique Dutra; *Hospital Assunção*: Silvia Regina Ramos, Lilian Mara Perroud Miilher; *BP - A Beneficência Portuguesa de São Paulo - Unidade Paulista*: Viviane Cordeiro Veiga, Salomón S. O. Rojas; *BP - A Beneficência Portuguesa de São Paulo - Unidade Mirante*: Fernando Jose da Silva Ramos, Cristiane Moretto Santoro; *Hospital Butantã*: Antônio C. A. Beneventi, Selma S. B. Silva; *Hospital de Base de São José do Rio Preto*: Suzana Margareth Lobo; *Hospital de Clínicas Caieiras*: Célia Matos, Bárbara Dell Ort; *HCor-Hospital do Coração*: Edson Renato Romano, André Franz da Costa, Jorge Alcantara Farran, Marcelo Luz Pereira Romano, Vinícius Avellar Werneck; *Hospital e Maternidade Brasil*: Sylas Bezerra Cappi, Ana Paula Mascarelli, Maira, Maurício Rocca, Herculano Dinis; *Hospital e Maternidade Ipiranga Arujá*: Rafael Di Domenico Mattos; *Hospital Guarulhos*: Fabio de Carvalho Mauricio; *Hospital Ipiranga*: Eder Donizetti Peres de Oliveira, Luiz Henrique Costa Garcia, Fernanda Rubia Negrão; *Hospital Israelita Albert Einstein*: Thiago Domingos Corrêa; *Hospital*

Madre Theodora: Átila Vendite Lourenço Pinheiro; *Hospital Nove de Julho*: Carlos Eduardo Nassif Moreira, Luiz Monteiro da Cruz Neto, Aquiles Oliveira Wanghon, Mariza D'Agostino Dias, Adriano da Silva Machado; *Hospital Novo Atibaia*: Rubens Sergio da Silva Franco, Amauri Francisco De Marchi Benfica, Walter Carlos Giradelli Baptista, Manoela Moreira de Sousa, Aline Ribeiro Moreira; *Hospital Paulistano*: Airton Leonardo de Oliveira Manoel, Ciro Parioto Neto, Israel Pinheiro Campos, Gustavo Ricci; *Hospital Samaritano Higienópolis*: Samantha Longhi Simões de Almeida, Lucas Junho Ricotta, Rosa Goldstein Alheira Rocha, Leonardo F. Ferrari Nogueira; *Hospital São Bernardo*: Fabio de Carvalho Mauricio; *Hospital São Francisco*: Marcus Antônio Ferez, Edson Antônio Nicolini, Geraldo da Silva Prado Neto, Franceliana Prado Barbosa Sgobi, Carlos Mencarini Pires Liberal; *Hospital São Paulo*, *Universidade Federal de São Paulo*: Flavia Ribeiro Machado, Flavio Geraldo Rezende Freitas; *Hospital SEPACO:* Flávio Geraldo Rezende de Freitas, Sérgio Sônego Fernandes, Tamiris Uracs de Sales Graça; *Hospital Sírio-Libanês*: Laerte Pastore Jr, Bruno Tomazini, Luciano Azevedo, Fernando Jose da Silva Ramos; *Hospital Vera Cruz*: Ronaldo Padovani; *Hospital Vivalle*: Fernando Vinicius Cesar De Marco

Uruguay

Montevideo - *CAMOC*: Gastón Burghi, Pedro Azulgaray, Dardo Gonzalez; *COMECA*: Gastón Burghi, Pedro Alzugaray; *COMERO IAMPP:* Andres Cebey, Nelson de lo Santos, Ines Quiroga; *Hospital Maciel:* Gastón Burghi, Eduardo Moreira; *Sanatorio Americano*: Gastón Burghi, Pedro Azulgaray, Dardo Gonzalez

The ORCHESTRA - Phase 4 (2022-2023)

Steering Committee: Marcio Soares, Fernando Augusto Bozza, Jorge Ibrain Figueira Salluh, Pedro Martins Pereira Kurtz, Gastón Burghi, Fernando Godinho Zampieri

Research Coordinators: Aline Reis da Silva Antunes, Grazielle Viana Ramos

Statisticians: Leonardo dos Santos Lourenco Bastos, Lunna Perdigão Borges, Thaís Machado, Gabriel Miranda

Investigators and participating centers

Brazil

Alagoas - *Hospital Memorial Arthur Ramos*: Maria Valéria de Carvalho Wanderley; Aline dos Santos Carvalho, Amanda Ribeiro de Mendonça Picone, Morghana Aparecida Rodrigues Ferreira, Bruna Xavier Brito; **Amazonas** - *Hospital Delphina Rinaldi Abdel Aziz*: Liane Cavalcante, Irina Jerez Jerez, Yudermys Amezaga Santana, Helena Alvarenga Sardenberg, Edna Freitas Martins, Marcia Lidiane Vasconcelos Dias Amorim; **Bahia** - *Hospital Cardiopulmonar*: Edson Silva Marques Filho; Antônio Fernando Borba Fróes Júnior, Daniel Beckerath da Silva Leitão; *Hospital Geral Cleriston Andrade*: Lúcio Couto de Oliveira Junior, Patrick Harrison Santana Sampaio, Renata Nunes de Oliveira, Diego Venicio Santos Argolo, Vanessa Freitas Vital, Bruno Cunha de Oliveira, João Victor Brito do Vale, Ramaiana de Jesus Gonzaga Cavalcante, Lúcio Couto de Oliveira Junior, Joaquim Agatângelo de Souza, Alberto Manoel Sarkis de Oliveira, Larissa Fernandes Oliveira, Daniela Cunha de Oliveira, Ricardo Peixoto Oliveira, Paulo Henrique Panelli Ferreira, Jamyllo Sales Brito, Elissama de Jesus Sena Reis, Vinicius Silva Oliveira, Geiza Santana Vidal; *Hospital da Cidade:* André Luiz Nunes Gobatto, Sydney Agareno de Souza Filho, Luciana Sampaio de Mattos Palmeira, Licurgo Pamplona Neto, Livia Magalhães Costa Castro; *Hospital Unimed Baia de Todos os Santos*: Lúcio Couto de Oliveira Junior; Joaquim Paulo Castro de Santana, Diego Venício Argolo, Patrick Harrison Sampaio, Joaquim Agatângelo Sousa, Tarsila Correia Ribeiro; **Ceará** - *Hospital Monte Klinikum*: David Theophilo Araújo, Victor Souza Cravo, Douglas Holanda Campos Filho, Ricardo Eustáquio Magalhães, Manuella Meireles Victor Souza Cravo, Pereira Gadelha Santos, Francisca Jane Gomes de Oliveira; **Distrito Federal** - *Hospital Anchieta*: Marcelo Oliveira Maia, Adriano Drummond, Noara Barros, Carla Moggia, Ivna Asfor; *Hospital Santa Luzia:* Marcelo Oliveira Maia, Fábio Amorim, Carlos Darwin; *Hospital DF Star*: Antônio Aurélio Fagundes Jr; *Hospital Brasília Unidade Águas Claras*: Vinicius Machado Santos, Pedro Henrique Rosa da Silveira, Tiago Samuel Lima Pontes, Flavio Carvalho dos Santos; **Espírito Santo** - *Hospital Meridional:* Marcus Vinicius A. Leitão, Lucas Resende Aniceto, Lucas Dornelas F. Machado Silva, Shayra Pansini Souza, Frederico Machado de Siqueira; *Hospital Unimed Vitória*: Eliana Bernardete Caser; **Goiás** - *Hospital Municipal de Aparecida de Goiânia*: Joan Rodrigues de Castro, Maurício Mascarenhas Boaventura, Cristian Andrade Garcia, Renata de Souza Cyrino, Gean Carlos Alves Moraes; *Hospital Israelita Albert Einstein - Goiânia*: Ângelo Antônio Gomes de Carvalho; *Centro Estadual de Reabilitação e Readaptação Dr. Henrique Santillo*: Eduardo Vilela, Ciro Bruno Silveira Costa, Priscila Martins Pereira, Ludmila Gomes dos Santos, Ronycley Rocha Rezende, Fabianne Silveira Cardoso; *Hospital*

Estadual de Urgências Governador Otávio Lage de Siqueira: Antônio Elias Lopes, Flávio Augusto Castro, Gustavo Prudente, Alex Linhares, Gabriel Fogaça, Igor Ferreira Capelleti; **Maranhão** - *Hospital São Domingos:* José Raimundo Azevedo, Luis Eduardo França Tupinambá Junior; *UDI Hospital*: Alexandre Guilherme Ribeiro de Carvalho, Edilene Coelho de Souza Novaes, Lucas Akira Costa Hirai, Daniel Wagner de Castro Lima Santos, Louise Aline Romão Gondim, Tânia Karla Sousa Nogueira Rosa; *Hospital Maranhense*: Filipe Sousa Amado; *Hospital do Cancer do Maranhão Tarquinio Lopes Filho*: Gustavo Teixeira Alves, Sara Vieira Nascimento; *Hospital de Traumatologia e Ortopedia do Maranhão*: Luciana Sousa Silva, Rosinete Andrade Ferreira; **Minas Gerais** - *Hospital Metropolitano Doutor Célio de Castro*: Luidy Luciano Cardoso, Roberto Sydney, Paolo Tótola, Bruno Resende, Diogo Madeira; *Hospital Felício Rocho*: Rogério de Castro Pereira, Thais de Paula Guimarães, Sinval Lins Silva, Daniel Fontes, Débora Avelar Afonso da Silva, Ana Clara Ferreira Amâncio Pereira; *Hospital das Clínicas da UFMG*: Saulo Fernandes Saturnino; *Pró-Saúde Hospital Metropolitano Vale do Aço*: Luiz Henrique de Araújo Pereira Costa, Norberto de Sá Neto, Rodrigo Silveira Machado, José Roberto Batista; *Hospital Risoleta Tolentino Neves*: Marco Aurélio Reis, Pulchéria Leôncio Pereira Araujo, Camila Martins Ramos; **Pará** - *Hospital Adventista de Belém*: Edgar de Brito Sobrinho; **Paraíba** - *Hospital Nossa Senhora das Neves*: Paulo César Gottardo, Elbia Assis Wanderley, Katyucia Egito de Araújo Urquisa, Andreia Cristina Fumagalli Cainelli, Vinilton Leandro Ferreira; *Hospital Universitário Lauro Wanderley*: Ciro Leite Mendes, Igor Mendonça do Nascimento; **Paraná** - *Hospital Santa Cruz*: Hipolito Carraro Jr; *Hospital São Marcelino Champagnat*: Viviane Bernardes de Oliveira Chaiben, Maria Lygia Minney, Andressa de Souza Bertoldi, Karen Fernandes de Moura, Gustavo Henrique dos Santos Silva, Bruno Alcantara Gabardo; *Hospital Municipal Padre Germano Lauck*: Roberto de Almeida, Guilherme Ribeiro, Mirian Liliana Insfrán Franco; *Hospital Universitário Cajuru*: Viviane Bernardes de Oliveira Chaiben, Giovanna Cerri Lessa, Victor Hugo Santana Lourenço de Lima, Leandro Bressianini Jurkonis, Marcos vinicius Streit, Gabriela Martins Teixeira; *Hospital Nossa Senhora das Graças*: Iara Buselato Chen, Vanessa Padilha Tomba, Marta Ângela Brandão; *Hospital Araucária de Londrina*: Cintia Magalhães Carvalho Grion; *Hospital Universitário Regional do Norte do Paraná*: Cintia Magalhães Carvalho Grion; **Pernambuco** - *Real Hospital Português*: Mário Diego Teles Correia; *Hospital Santa Joana Recife*, Gustavo Trindade Henriques Filho, Arthur Henrique Ribeiro do Valle de Faria, Marcos Antonio Cavalcanti Gallindo, Carlos Eduardo Ferraz Freitas, Ana Flávia de Melo Campos, Danielle Ferraz de Oliveira Aguiar; **Rio de Janeiro** - *Hospital Copa D'Or*: Aloysio Saulo Beiler; *Hospital Quinta D'Or*: Alexandre Coscia, Laura Herranz Prinz, Juliana Gurgel da Silveira, Cristiane Belo, Soraya Pulier, Joao Vitor Bessa, Juliana Gravina, Thiago Prata, Joyce Roma Lucas de Silva, Alessandra Longo, Lorena fonseca, Alexandra Gonçalves, Marcelo Cruzick; *Hospital Estadual Alberto Torres*: Ulisses de Oliveira Melo; *Hospital Nossa Senhora do Carmo*: Paula Figueiredo Natel, Raphaela Mannarino, Nelson Poubel, Amir Gonçalves Neto, Marcelo Motta; *Complexo Américas*: Victor Cravo, Emmanuel Salgueiro; *Hospital Norte D'Or*: Douglas Quintanilha Braga, Sergio Teixeira Sant'Anna Júnior, Gustavo Caniné da Costa, Renata Ribeiro Leite do Amaral, Camila Lima Ferreira da Costa, Thalita Montenegro Prieto Lloret; *Hospital Barra D´Or*: Walter Homena Jr, Marcelo de Sousa Santino, Gloria Adriana Rocha Martins, José Alexandre Espósito Panaro, Francisco Gonçalves Gabriel, Paula Gorgulho, Marcelo Felix, Luciana Freitas de Oliveira; *Hospital Pasteur*: Pedro Henrique Barbosa D' Almeida, Carlos Eduardo Brandão, Raquel Pereira de Farias Evangelista, Danessa Moreira Rodrigues; *Hospital Rios D'Or*: Alessandra Alves; *Hospital Caxias D'Or*: Eric Perecmanis; *Hospital Niterói D'Or*: Ricardo Turon Bruno Guimarães; *Hospital Oeste D'Or*: Guilherme Brenande Alves Faria, Márcia Adélia de Magalhães Menezes, Liliane Rodrigues de Mendonça, Reinaldo Campos Rodrigues, Caros Henrique Ferreira Ramos, Rosa Imaculada Stancato, Joyce Andrade; *Hospital São Lucas Copacabana*: Aline Affonso, Laura Herranz Prinz, Bruno Gonçalves; *Hospital Municipal Evandro Freire*: Fabio Basilio Fernandes dos Santos; *Hospital Adventista Silvestre*: Fernando Santiago Montenegro; *Hospital Badim*: Edmundo de Oliveira Tommasi, Alexandra Goncalves da Silva, Alexandre Vaz Scotti, Jefferson dos Santos Daros, Fábio Guilherme Santoro; *Hospital Samer:* Henrique Miller Balieiro; Marcelo Namen; *Hospital Copa Star:* Pedro Kurtz, Rodolfo Espinoza, Rafaella Pottes, *Hospital de Clínicas de Jacarepaguá*: Fernando Alves Rocha, Simone Cristina Santos de Lira, Ana Paula Figueiredo de Carvalho, Carlos Eduardo Brandão; *Hospital Gloria D'Or*: Cecília Magno; Nathane Santanna; *Hospital Municipal da Japuiba*: Viviane Bogado Leite Torres, Michelle Cristina Ferreira Soares, Beatriz Victoria Correia Ferreira, Josiane Cristina da Silva, Julia Monteiro Novaes, Tatiany Lopes Lessa; *Hospital de Clínicas Mario Lioni*: Fernando Santana Pinto, Carlos Eduardo Brandão, Juliana Sales de Ornelas; *Hospital e Maternidade Santa Lúcia*: Eduardo Costa Pinto, Carlos Eduardo Brandão, Elen Silva Ferreira, Marcelo Alonso de

Barros Correia; *Hospital da Fundação Eletronuclear de Assistência Médica*: Viviane Bogado Leite Torres, Mariana Rodrigues Farias Andrade; *Instituto Nacional de Câncer - Hospital do Câncer II*: Rodolfo Espinoza, Rafael Mandarino; **Rio Grande do Sul** - *Hospital Mãe de Deus*: Lucas Vieira de Souza; *Hospital de Clínicas de Porto Alegre*: Fabiano Márcio Nagel, Márcio Manozzo Boniatti; *Hospital Dom João Becker*: Michael Milman; **Santa Catarina** - *Hospital Municipal Ruth Cardoso*: Pedro Salomão Dias, Cesar Augusto Meirelles de Almeida, Eduardo Bellotto, Pablo Wanglon Richter; **São Paulo** - *Hospital A.C. Camargo*: Antônio Paulo Nassar Jr, Silvana Soares dos Santos; *Hospital e Maternidade Brasil*: Fabio de Carvalho Mauricio, Tatiana Gozzi Pancev Toledo, Fernando Ramos Pellegrini, Manoela Prado Pasqualucci Esposito; *Hospital Municipal Moysés Deutsch*: Niklas Soderberg Campos, Bruna Achar Soderberg Campos, Paula Geraldes David João, Luiz Adriano Esteves, Petrus Söderberg Campos, Luciana Silveira de Oliveira; *Hospital Nove de Julho*: Marcos Soares Tavares, Celso Madeira Padovesi, Antônio Paulo Martins Ramos Filho; *Hospital do Servidor Público Estadual, IAMSPE*: Ederlon Alves de Carvalho Rezende, Ellen Pierre Oliveira, Caio Gouvêa Jaoude, Vânia Quinato Malacize, Mateus Demarchi Gonsalves; *Hospital de Base*: Suzana Ajeje Margareth Lobo, Luana Fernandes Machado, Juliana Devós Syrio Martinez, Marcio Mussolino de Queiroz, Neymar Elias de Oliveira, Silvia Prado Minhoto Teixeira Ramin, Vanessa Aparecida Maziero Santana; *Hospital São Luiz Morumbi*: José Célio Vieira Brandão, Adriana Peixoto Gelmetti de Barros; *Hospital Vivalle*: Flavio Rodrigues de Sousa, Felipe de Jesus Gonçalves; *Hospital São Luiz Itaim*: Mariza Silva Ramos Loesch, Thiago Gomes Romano; *Hospital Villa-Lobos*: Carlos Antônio Carvalho Ribeiro, Flávio Geraldo Rezende de Freitas; *Hospital Israelita Albert Einstein - Morumbi*: Thiago Domingos Corrêa; *HCor-Hospital do Coração*: Edson Renato Romano, Rosianne Vasconcelos; *Hospital Assunção*: Silvia Regina Ramos; *Hospital Metropolitano*: Eduardo Augusto Pessoa Gomes, Carlos Eduardo Brandão, Liliane Alves Feitoza Turci, Aline Nunes Lobo Bueno; *Hospital da Luz*: Daniel Almeida Schettini, Carlos Eduardo Brandão, Vaneska Mazzini, Lilian Louise Coelho Pereira; *Hospital Sírio Libanês - Bela Vista:* Laerte Pastore Jr, Fernando José da Silva Ramos, Naira Lima Matos, Clara Esther Maciel dos Santos, Bruno Martins Tomazini, Nilda Rosa de Oliveira Prado; *Hospital Panamericano*: Eduardo Costa Pinto, Carlos Eduardo Brandão, Vanessa Ribeiro Pardauil, Cristiane Cunha da Silva Rosa; *Hospital Vitória Anália Franco*: Guilherme Rossini, Carlos Eduardo Brandão, Mariana Celeghini Santiago Gosik, Priscilla Belchior Gonzalez; *Serviço Social da Indústria do Papel Papelão e Cortiça do Estado de São Paulo - Hospital e Maternidade SEPACO*: Nathaly Fonseca Nunes, Daniela Boschetti, Flávio Geraldo Rezende de Freitas; *Hospital Alvorada Moema*: Amanda Mota de O. Veiga, Carlos Eduardo Brandão; *Hospital Carlos Chagas*: Fernando Jose Bricks, Carlos Eduardo Brandão, Caroline Scodelario Cortes, Francisco Afranio Miranda; *Hospital São Paulo*: Flavia Ribeiro Machado; *Hospital Santa Helena*: Alessandra Borges Mendes Gonzaga, Carlos Eduardo Brandão, Tatiane Oliveira Luiz, Liliane Lemos; *Hospital Japonês Santa Cruz*: Thiago Miranda Lopes de Almeida, Sérgio Sônego Fernandes, Carlos Antônio Carvalho Ribeiro, Flávio Geraldo Rezende de Freitas; *Hospital Samaritano Higienópolis*: Marcos Cairo Vilela, Barbara Cristina de Abreu Pereira, Fernando Antonio Alvares da Costa, Leonardo Fernando Ferrari Nogueira, Natalia Lopes Ferreira, Luciana Rosa Fidelis; *BP - A Beneficência Portuguesa de São Paulo - Unidade Mirante*: Viviane Cordeiro Veiga; *Hospital Ana Costa*: Fernanda Rodrigues Martins Masteguim, Carlos Eduardo Brandão, Thiago Santos da Silva, Fatima Cristina Andrade Rodrigues; *Hospital Paulistano*: Alder Costa Garcia da Silveira; Carlos Eduardo Brandão, Debora Prudencio e Silva, Helenice de Paula Vieira; *Santa Casa de Piracicaba*: Rafael Angelo Tineli, Luciana Marcolino Tineli; *Hospital Beneficência Portuguesa de Ribeirão Preto*: Marcus Antônio Ferez; Franceliana Prado Barbosa Sgobi, Fernanda Valéria Ramos Paiolo; *Hospital e Maternidade Ribeirão Pires*: Fabio de Carvalho Mauricio, Tatiana Gozzi Pancev Toledo, Fernando Ramos Pellegrini, Manoela Prado Pasqualucci Esposito; *Hospital dos Fornecedores de Cana de Piracicaba*: Renata Lopes Basso, Rafael Angelo Tineli; *Hospital Pitangueiras*: Márcio Shimabuku e Silva, Carlos Eduardo Brandão, Felipe Neves Marcelino, Alex Oliveira; *Hospital Vila Nova Star*: Thiago Gomes Romano; *Hospital São Bernardo*: Ana Paula Mascarelli Amaral, Carlos Eduardo Brandão, Maria de Fatima Silva de Almeida, Janaina de Cassia Lopes dos Santos; *Hospital Novo Atibaia*: Rubens Sergio da Silva Franco, Amauri Francisco de Marchi Bemfica, Walter Carlos Girardelli Baptista, Manoela Moreira de Sousa, Aline Ribeiro Moreira, Juliana Regina Berto Wada; *Hospital de Clinicas de Caieiras*: Volnei Martins Castanho, Carlos Eduardo Brandão, Priscila Gonzaga dos Santos, Elaine Santana da Silva, Marciely Alves Ramalho de Oliveira, Viviane Gimenez Rossini, Imara Jacinto de Azevedo Rios; *Hospital Ipiranga*: Rafael Di Domenico Mattos, Carlos Eduardo Brandão, Fabricio Campos Morais Moreira, Julienne Garcia Rissatti, Luiz Henrique Costa Garcia; *Hospital Santo Amaro*: Sanmya Danielle Rodrigues dos Santos, Carlos Eduardo Brandão, Dominique Almeida

Cruz, Bárbara Fialdini Von Ah; *Hospital e Maternidade Ipiranga Arujá*: Rafael Di Domenico Mattos, Carlos Eduardo Brandão, Gisele Aparecida Cardoso; *Hospital das Clínicas da Faculdade de Medicina da Universidade de São Paulo*: Leandro Utino Taniguchi, Bruno Adler Maccagnan Pinheiro Besen, Roberta Muriel Longo Roepke, Pedro Fortes Osório Bustamante, Ana Clara Marcondes Dobre; *Unimed Capivari*: Rafael Angelo Tineli, Daniela Mazzini Quagliato Azanha; *Hospital IFOR*: Sílvia Regina Ramos

Uruguay

Montevideo - *Hospital Maciel*: Gastón Burghi; *Sanatório Americano*: Gastón Burghi, Pedro Alzugaray; *COMECA*: Gastón Burghi, Carlos Pan; *Hospital Policial*: Pedro Saldun, Gonzalo Lacuesta, Sergio Rovira, Lourdes Ferro, Silvana Lopez; *Hospital Evangélico*: Gastón Burghi; *COMERO IAMPP*: Andrés Cebey, Carlos Cardoso; *CRAMI*: Gastón Burghi, Pedro Alzugaray; *CAMOC*; Gastón Burghi, Pedro Alzugaray

APPENDIX 2S - METHODS (*ACCORDING TO THE MOST RECENT STUDY PHASE 4)

Design and Setting

The ORCHESTRA study is a multicenter retrospective cohort study of critical care organization and outcomes performed in Brazil and Uruguay. The study is coordinated by the Department of Critical Care of the *Instituto D'Or de Pesquisa e Ensino* (IDOR), Rio de Janeiro, Brazil, and endorsed by the Brazilian Research in Intensive Care Network (BRICNet),[13] an independent research network for performing investigator-initiated multicenter studies in critical care in Brazil. The Brazilian National Ethics Committee (Brazil CAAE: 19687113.8.1001.5249) and the Ethics Committee of the *Hospital Maciel*, Montevideo, Uruguay (protocol number 20/2017) approved the study and waived the need for informed consent.

Selection of centers

We restrict the study to ICUs registered in the BRICNet database with ≥ 8 beds and known to use the Epimed Monitor System®, (Epimed Solutions®, Rio de Janeiro, Brazil), a commercial cloud-based registry for quality improvement, performance evaluation and benchmarking purposes, for at least one calendar year.[14] ICUs are invited to participate in the study and those accepting the invitation are asked to complete a survey with information on the respective ICU and hospital, and to extract patients' data from the Epimed Monitor® system.

Data collection and definitions

Intensive care unit and hospital data

In each participating ICU, the ICU director and/or chief nurse complete an online survey constructed in Research Electronic Data Capture (REDCap)[15] application about hospital and ICU characterization and organizational, structural and process characteristics. Domains of the survey are based on prior studies demonstrating potential structure-outcome links in critical care revised considering the current literature at each phase, and include hospital and ICU characterization, ICU and hospital structure, presence of certified training programs in critical care, ICU staffing patterns, multidisciplinary clinical rounds, checklists, implementation of clinical protocols to prevent healthcare- associated complications, level of autonomy of non-physician care providers to take decisions at the bedside, and family visiting policies.

We define board-certified intensivists and nurses as those who were board-certified in Intensive Care Medicine by critical care societies in each participating country. For the purposes of the present study, we consider only protocols fully implemented when they are in use for at least six months in the ICU.

In order to guarantee data accuracy, validity and completeness, in each phase, we pilot the survey among three participating centers, providing respondents with explicit definitions of the survey domains. We also ensure the reliability and consistency by reviewing of all data by interviews with medical and/or nurse ICU directors at every participating center by phone or web conference.

Patients' data

We ask investigators in each center to extract deidentified patients' data from the Epimed Monitor System®. Data are prospectively entered in a structured electronic case report form by a combination of data integrated with local electronic health records and data entered manually by trained case manager (in general, a nurse). Online or live training and feedback sessions are held regularly in addition to at least six face-to-face meetings per year, during which users receive updates and additional training.

The Epimed Monitor System® is structured to have active controls to guarantee data quality and checking. There are no free text fields; all variables are structured with internal linked codes. To minimize processing errors, which encompasses coding and data entry steps, the definitions/labels of each variable are clearly stated in the eCRF and are also available in a PDF sheet easily accessible in the online platform. To address possible errors, the system provides checking during data entry process ("interactive checking"). Conditional filling is also present for some specific variables (eg. diagnoses, pathogens and antibiotics). For physiological and laboratory data, values beyond the usual range for the variable are highlighted for rechecking and implausible values cannot be recorded. Intensive care units coordinators and case managers can monitor incomplete cases and check for the percentage of incompleteness of specific variables over a period. In addition, offline checks can occur at random, depending on demand for each unit and for database updating and improvement.

We include all consecutive patients aged ≥ 16 years old admitted to the participating ICUs during study phase period. Readmissions and patients with missing core data (age, location before ICU admission, main ICU

admission diagnosis, the Simplified Acute Physiology Score (SAPS) 3),[16] ICU and hospital length of stay (LOS) and vital status at hospital discharge) are excluded. In addition, ICUs with more than 10% missing core data are also excluded.

Patients' data elements include demographics, comorbidities based on the Charlson Comorbidity Index,[17] functional status in the week before hospital admission adapted from the Eastern Cooperative Oncology Group (ECOG) performance status,[4] frailty based on the Modified Frailty Index,[18] location before ICU admission, scores including the SAPS 3[16] and the Sequential Organ Failure (SOFA),[19] ICU admission diagnosis, use of ICU support, laboratory and physiological data at admission, ICU and hospital LOS and destination after hospital discharge.

Outcomes

The primary outcome of interest is in-hospital mortality at the patient level. The secondary outcomes are the ICU mortality, ICU and hospital LOS. We also evaluate the ICUs' clinical performance by estimating the standardized mortality rates (SMR) and efficiency in resource by estimating the standardized resource use (SRU) according to the SAPS 3, as proposed by Rothen et al.[20,21] We calculate the SMR by dividing observed by predicted mortality rates. The SRU estimates the average observed to expected ratio of resources (based on the length of ICU stay) used per surviving patient in a specific ICU adjusted for the severity of illness. Based on median SMR and median SRU, we assigned each unit to one of four groups: "most efficient" (all units whose SMR and SRU were below the median SMR and SRU); "least efficient" (units with both SMR and SRU above the median); "overachieving" (low SMR and high SRU) and "underachieving" (high SMR and low SRU).[20]

Data processing and missing variables

Patients' and centers' deidentified data are centrally processed and analyzed in dedicated servers with control of accesses and logs in compliance with data privacy and protection regulations (e.g. LGPD - *Lei Geral de Proteção de Dados Pessoais* in Brazil). We screen ICU and hospital data for missing information, implausible and outlying values, logical errors and insufficient details. We impute non-core data missing less than 1% of values using the most frequent category; otherwise, we use a multiple imputation technique. We do not impute missing SAPS 3 directly; instead, when possible, we attribute the missing values to individual SAPS 3 components and then recalculate the score. The statistical analyses are described in the scope of each specific study.

Table 1S - Summary of published articles using the ORCHESTRA databases

Title	Purpose	Hospitals/ ICUs/patients (n)	Main findings
Organizational characteristics, outcomes, and resource use in 78 Brazilian intensive care units: the ORCHESTRA study[1]	To investigate the impact of organizational factors on the outcomes and resource use in a large sample of Brazilian ICUs.	Hospitals: 51 ICUs: 78 Patients: 59,693	The number of protocols was the only organizational characteristic independently associated with mortality (OR = 0.944; 95%CI 0.904 - 0.987). The effects of protocols were consistent across subgroups including surgical and medical patients as well as the SAPS 3 tertiles. A significant trend toward efficient resource use as the number of protocols increased was also observed.
Effects of organizational characteristics on outcomes and resource use in patients with cancer admitted to intensive care units[2]	To investigate the impact of organizational characteristics and processes of care on hospital mortality and resource use in patients with cancer admitted to ICUs.	Hospitals: 49 ICUs: 70 Patients: 9,946	Dedicated clinical pharmacists in the ICU (OR = 0.67; 95%CI 0.49 - 0.90), number of protocols (OR 0.92; 95%CI 0.87 - 0.98), and daily meetings between oncologists and intensivists for care planning (OR 0.69; 95%CI 0.52 - 0.91) were independently associated with lower mortality. Implementation of protocols (OR 1.52; 95%CI 1.11 - 2.07) and meetings between oncologists and intensivists (OR 4.70; 95%CI 1.15 - 19.22) were also independently associated with more efficient resource use. Neither admission to ICUs in cancer centers compared with general hospitals nor annual case volume had an impact on mortality or resource use.
External validation of SAPS 3 and MPM_0-III scores in 48,816 patients from 72 Brazilian[3]	To validate the SAPS 3 and MPM_0-III scores in a large contemporary cohort of patients admitted to Brazilian ICUs.	Hospitals: 50 ICUs: 72 Patients: 48,816	SMRs obtained for each model were: 1.00 (95%CI 0.98 - 0.102) for the SAPS 3-SE, 0.75 (0.74 - 0.77) for the SAPS 3-CSA and 1.15 (1.13 - 1.18) for the MPM_0-III. Discrimination was better for SAPS 3 models (AUROC = 0.85) than for MPM_0-III (AUROC = 0.80) ($p < 0.001$). In calibration analysis, the SAPS 3-CSA overestimated mortality throughout all risk classes while the MPM_0- III underestimated it uniformly. The SAPS 3-SE did not show relevant deviations from ideal calibration.
The effects of performance status one week before hospital admission on the outcomes of critically ill patients[4]	To assess the impact of performance status impairment one week before hospital admission on the outcomes in patients admitted to ICUs.	Hospitals: 51 ICUs: 78 Patients: 59,693	Performance status impairment was moderate in 17.3% and severe in 6.9% of patients. Overall, the worse the performance status, the higher the ICU and hospital mortality and LOS. In addition, patients with worse performance status were less frequently discharged home. Performance status impairment was associated with worse outcomes in all SAPS 3, Charlson Comorbidity Index and age quartiles as well as according to the admission type. Adjusting for other relevant clinical characteristics, performance status impairment was associated with higher hospital mortality (OR = 1.96; 95%CI 1.63 - 2.35), for moderate and OR = 4.22 (3.32 - 5.35), for severe impairment.
Family care, visiting policies, ICU performance, and efficiency in resource use: insights from the ORCHESTRA study[5]	To explore the association between family care-related policies and ICU performance and resource use adjusted for the severity of illness.	Hospitals: 51 ICUs: 78 Patients: 59,693	Median visiting hours was 2 (1.5 - 6) and only 3 (4%) ICUs had unrestricted open visiting policies. In general, ICUs with better clinical performance had more liberal policies and more frequently implemented processes to improve communication with families in different domains. The same was observed in ICUs with a high efficiency in resource use. In addition, visiting hours were higher in high-efficiency ICUs [4 (2 - 7) *versus* 2 (1 - 4), p = 0.001].
Role of organizational factors on the 'weekend effect' in critically ill patients in Brazil: a retrospective cohort analysis[6]	To investigate the effects of ICU organizational and staffing patterns on the potential association between weekend admission and outcomes in critically ill patients.	Hospitals: 51 ICUs: 78 Patients: 59,693	Weekend admissions were not associated with higher hospital mortality (OR 1.05; 95%CI 0.99 - 1.12; p = 0.095). However, a 'weekend effect' was still observed in scheduled surgical admissions, as well as in ICUs not using checklists during the weekends. For unscheduled admissions, no 'weekend effect' was observed regardless of ICU's characteristics. For scheduled surgical admissions, a 'weekend effect' was present only in ICUs with a low number of implemented protocols and those with a reduction in the nurse/bed ratio and not applying checklists during weekends.
Association of frailty with short-term outcomes, organ support and resource use in critically ill patients[7]	To assess the association of frailty, measured by the Modified Frailty Index, with short-term outcomes and organ support used by critically ill patients.	Hospitals: 55 ICUs: 93 Patients: 129,680	There were 18.9% frail (MFI ≥ 3) patients. Frailty was associated with higher in-hospital mortality (OR 2.42; 95%CI 1.89 - 3.08), particularly in patients admitted with lower SOFA scores. Frail patients were less likely to be discharged home (OR 0.36; 95%CI 0.54 - 0.79) and had higher hospital and ICU LOS than non-frail patients. Use of all forms of organ support (mechanical ventilation, non-invasive ventilation, vasopressors, dialysis and transfusions) were more common in frail patients and increased as MFI increased.
ICU staffing feature phenotypes and their relationship with patients' outcomes: an unsupervised machine learning analysis[8]	To study whether ICU staffing features are associated with improved hospital mortality, ICU LOS and duration of MV using cluster analysis directed by machine learning.	Hospitals: 55 ICUs: 93 Patients: 129,680	Three clusters were identified. The features distinguishing the clusters were the presence of board-certified intensivists in the ICU 24/7 (present in Cluster 3), dedicated pharmacists (present in Clusters 2 and 3) and the level of nurse autonomy (which increased from Clusters 1 to 3). The patients in Cluster 3 exhibited the best outcomes, with lower adjusted hospital mortality (OR 0.92; 95%CI 0.87 - 0.98), shorter ICU LOS [SHR for patients surviving to ICU discharge 1.24 (95%CI 1.22 - 1.26)] and shorter durations of MV [SHR for undergoing extubation 1.61(95%CI 1.54 -1.69)]. Cluster 1 had the worst outcomes.

Continue...

...continuation

Title	Purpose	Hospitals/ ICUs/patients (n)	Main findings
Modulators of systemic inflammatory response syndrome presence in patients admitted to intensive care units with acute infection: a Bayesian network approach[9]	To investigate whether some confounding factors namely age, sex, performance status, source of infection and presence of diabetes and active cancer, could modulate the presence of SIRS at intensive care unit admission.	Hospitals: 55<br>ICUs: 93<br>Patients: 129,680	After adjusting for specified variables, age (OR 0.90; 95%CI 0.89 - 0.91, for 10-year increment), cancer (OR 1.40; 95%CI 1.31 - 1.50), performance status (OR 1.21; 95%CI 1.14 - 1.29 for moderate impairment and OR 1.26; 95%CI 1.18 -1.36 for severe impairment) and infection source were associated with SIRS. Predicted probability of SIRS.
A comparison of mortality from sepsis in Brazil and England: the impact of heterogeneity in general and sepsis-specific patient characteristics[10]	To test whether differences in both general and sepsis-specific patient characteristics explain the observed differences in sepsis mortality between countries, using two national critical care (ICU) databases.	Brazil<br>ICUs: 62<br>Patients: 4,505<br><br>United Kingdom<br>ICUs: 164<br>Patients: 17,921	Crude hospital mortality was comparable (Brazil 41.4% *versus* England 39.3%; OR 1.12 [0.98 - 1.30]). After adjusting for general patient characteristics, there was an important change in the point-estimate of the OR (0.88 [0.75 - 1.02]). However, after adjusting for sepsis specific patient characteristics, the direction of effect reversed again with Brazil having higher risk-adjusted mortality (OR 1.22 [1.05 - 1.43]).
Customization and external validation of the Simplified Mortality Score for the Intensive Care Unit (SMS-ICU) in Brazilian critically ill patients[11]	To customize and externally validate the recently proposed Simplified Mortality Score for the ICU (SMS-ICU, a simple score for 90-daymortality that has no need for ancillary testing results) for in-hospital mortality and to compare its performance to SAPS 3.	Hospitals: 51<br>ICUs: 78<br>Patients: 43,017	In the validation cohort, median SMS-ICU was 13 (IQR 8 - 16) points and median SAPS 3 was 44 (IQR 36 - 51) points. Discrimination of SMS-ICU was good (AUROC 0.817; 95%CI 0.814 - 0.819) but slightly lower than of SAPS 3 (AUROC 0.845; 95%CI 0.843 - 0.848;). The customized SMS-ICU predictions were comparable to SAPS 3 in terms of calibration. SMS-ICU can be used as a measure of illness severity for acutely admitted ICU patients in clinical studies. It is conceivable that overall performance of the SIRS criteria as a screening tool may be different depending on the population, its risk factors and age, among other confounders.
Comparing continuous versus categorical measures to assess and benchmark intensive care unit performance[12]	To compare categorical and continuous combinations of the SMR and SRU to evaluate ICU performance.	Brazil/Uruguay<br>ICUs: 128<br>Patients: 277,459<br><br>The Netherlands<br>ICUs: 83<br>Patients: 164,399	The SMR and SRU were more correlated in Brazilian/Uruguayan ICUs than in Dutch ICUs (Spearman's Rho: 0.54 *versus* 0.24). The highest and lowest ASER values were concentrated in the least and most efficient groups. An expert focus group listed potential advantages and limitations of both combinations.

ICU - intensive care unit; OR - odds-ratio; 95%CI - 95% confidence interval; SAPS - Simplified Acute Physiology Score; MPM - Mortality Probability Models; SMR - standardized mortality ratio; CSA - equation for Central and South American; AUROC - area under receiver operating characteristic curve; SE - standard equation; MFI - Modified Frailty Index; SOFA - Sequential Organ Failure Score; LOS - length of stay; MV - mechanical ventilation; SHR - sub-hazard ratio; SIRS - systemic inflammatory response syndrome; SMS - Simplified Mortality Score; IQR - interquartile range; SRU - standardized resource use; ASER - average standardized efficiency ratio.

REFERENCES

1. Soares M, Bozza FA, Angus DC, Japiassú AM, Viana WN, Costa R, et al. Organizational characteristics, outcomes, and resource use in 78 Brazilian intensive care units: the ORCHESTRA study. Intensive Care Med. 2015;41(12):2149-60.
2. Soares M, Bozza FA, Azevedo LC, Silva UV, Corrêa TD, Colombari F, et al, Effects of organizational characteristics on outcomes and resource use in patients with cancer admitted to intensive care units. J Clin Oncol. 2016;34(27):3315-24.
3. Moralez GM, Rabello LS, Lisboa TC, Lima MD, Hatum RM, De Marco FV, Alves A, Pinto JE, de Araújo HB, Ramos GV, Silva AR, Fernandes GC, Faria GB, Mendes CL, Ramos Filho RA, de Souza VP, do Brasil PE, Bozza FA, Salluh JI, Soares M; ORCHESTRA Study Investigators. External validation of SAPS 3 and MPM0-III scores in 48,816 patients from 72 Brazilian ICUs. Ann Intensive Care. 2017;7(1):53.
4. Zampieri FG, Bozza FA, Moralez GM, Mazza DD, Scotti AV, Santino MS, et al. The effects of performance status one week before hospital admission on the outcomes of critically ill patients. Intensive Care Med. 2017;43(1):39-47.
5. Soares M, Silva UV, Homena WS Jr, Fernandes GC, De Moraes AP, Brauer L, Lima MF, De Marco FV, Bozza FA, Salluh JI; ORCHESTRA (ORganizational CHaractEeriSTics in cRitcal cAre) Study Investigators. Family care, visiting policies, ICU performance, and efficiency in resource use: insights from the ORCHESTRA study. Intensive Care Med. 2017;43(4):590-1.
6. Zampieri FG, Lisboa TC, Correa TD, Bozza FA, Ferez M, Fernandes HS, et al. Role of organisational factors on the "weekend effect" in critically ill patients in Brazil: a retrospective cohort analysis. BMJ Open. 2018;8(1):e018541.
7. Zampieri FG, Iwashyna TJ, Viglianti EM, Taniguchi LU, Viana WN, Costa R, Corrêa TD, Moreira CE, Maia MO, Moralez GM, Lisboa T, Ferez MA, Freitas CE, de Carvalho CB, Mazza BF, Lima MF, Ramos GV, Silva AR, Bozza FA, Salluh JI, Soares M; ORCHESTRA Study Investigators. Association of frailty with short-term outcomes, organ support and resource use in critically ill patients. Intensive Care Med. 2018;44(9):1512-20.
8. Zampieri FG, Salluh JI, Azevedo LC, Kahn JM, Damiani LP, Borges LP, Viana WN, Costa R, Corrêa TD, Araya DE, Maia MO, Ferez MA, Carvalho AG, Knibel MF, Melo UO, Santino MS, Lisboa T, Caser EB, Besen BA, Bozza FA, Angus DC, Soares M; ORCHESTRA Study Investigators. ICU staffing feature phenotypes and their relationship with patients' outcomes: an unsupervised machine learning analysis. Intensive Care Med. 2019;45(11):1599-607.
9. Zampieri FG, Aguiar FJ, Bozza FA, Salluh JIF, Soares M; ORCHESTRA Study Investigators. Modulators of systemic inflammatory response syndrome presence in patients admitted to intensive care units with acute infection: a Bayesian network approach. Intensive Care Med. 2019;45(8):1156-8.
10. Ranzani OT, Shankar-Hari M, Harrison DA, Rabello LS, Salluh JI, Rowan KM, et al. A comparison of mortality from sepsis in Brazil and England: the impact of heterogeneity in general and sepsis-specific patient characteristics. Crit Care Med. 2019;47(1):76-84.
11. Zampieri FG, Granholm A, Møller MH, Scotti AV, Alves A, Cabral MM, et al. Customization and external validation of the Simplified Mortality Score for the Intensive Care Unit (SMS-ICU) in Brazilian critically ill patients. J Crit Care. 2020;59:94-100.
12. Bastos LS, Wortel SA, de Keizer NF, Bakhshi-Raiez F, Salluh JI, Dongelmans DA, et al. Comparing continuous versus categorical measures to assess and benchmark intensive care unit performance. J Crit Care. 2022;70:154063.
13. [BRICNet, a collaborative brazilian network to conduct and to promote multicenter studies in intensive care]. Rev Bras Ter Intensiva. 2007;19(3):408. Portuguese.
14. Zampieri FG, Soares M, Borges LP, Salluh JI, Ranzani OT. The Epimed Monitor ICU Database: a cloud-based national registry for adult intensive care unit patients in Brazil. Rev Bras Ter Intensiva. 2017;29(4):418-26.
15. Harris PA, Taylor R, Thielke R, Payne J, Gonzalez N, Conde JG. Research electronic data capture (REDCap)--a metadata-driven methodology and workflow process for providing translational research informatics support. J Biomed Inform. 2009;42(2):377-81.
16. Moreno RP, Metnitz PG, Almeida E, Jordan B, Bauer P, Campos RA, Iapichino G, Edbrooke D, Capuzzo M, Le Gall JR; SAPS 3 Investigators. SAPS 3--From evaluation of the patient to evaluation of the intensive care unit. Part 2: Development of a prognostic model for hospital mortality at ICU admission. Intensive Care Med. 2005;31(10):1345-55.
17. Charlson ME, Pompei P, Ales KL, MacKenzie CR. A new method of classifying prognostic comorbidity in longitudinal studies: development and validation. J Chronic Dis. 1987;40(5):373-83.
18. Zampieri FG, Iwashyna TJ, Viglianti EM, Taniguchi LU, Viana WN, Costa R, Corrêa TD, Moreira CE, Maia MO, Moralez GM, Lisboa T, Ferez MA, Freitas CE, de Carvalho CB, Mazza BF, Lima MF, Ramos GV, Silva AR, Bozza FA, Salluh JI, Soares M; ORCHESTRA Study Investigators. Association of frailty with short-term outcomes, organ support and resource use in critically ill patients. Intensive Care Med. 2018;44(9):1512-20.
19. Vincent JL, Moreno R, Takala J, Willatts S, De Mendonça A, Bruining H, et al. The SOFA (Sepsis-related Organ Failure Assessment) score to describe organ dysfunction/failure. On behalf of the Working Group on Sepsis-Related Problems of the European Society of Intensive Care Medicine. Intensive Care Med. 1996;22(7):707-10.
20. Rothen HU, Stricker K, Einfalt J, Bauer P, Metnitz PG, Moreno RP, et al. Variability in outcome and resource use in intensive care units. Intensive Care Med. 2007;33(8):1329-36.
21. Salluh JI, Soares M. ICU severity of illness scores: APACHE, SAPS and MPM. Curr Opin Crit Care. 2014;20(5):557-65.
