# Supplementary material for: A decade of the ORCHESTRA study: organizational characteristics, patient outcomes, performance and efficiency in critical care
Source: Crit Care Sci. 2024 Jul 1;36:e20240118en. doi: 10.62675/2965-2774.20240118-en (PMC11239203; doi:10.62675/2965-2774.20240118-en)
Supplement: Translation

# Translation: Uma década do estudo ORCHESTRA: características organizacionais, desfechos dos pacientes, desempenho e eficiência no cuidado intensivo

**DOI:** 10.62675/2965-2774.20240118-pt

**Authors:** Marcio Soares, Jorge Ibrain Figueira Salluh, Fernando Godinho Zampieri, Fernando Augusto Bozza, Pedro Martins Pereira Kurtz

**Affiliations:** 1 Brasil Instituto D’Or de Pesquisa e Ensino - Rio de Janeiro (RJ), Brasil.; 2 Canadá Faculty of Medicine and Dentistry, University of Alberta - Edmonton, Canadá.

## INTRODUÇÃO

A organização e a estrutura das unidades de terapia intensiva (UTIs) afetam a qualidade e a eficiência no cuidado intensivo.^(1,2)^ Como os cuidados agudos variam significativamente entre os países, as populações de pacientes e as práticas locais de cuidado, espera-se que as associações entre a estrutura, o processo e os desfechos nas UTIs também sejam diferentes dependendo do contexto. Atualmente, a maior parte da informação disponível sobre UTIs é proveniente de estudos realizados em países desenvolvidos, e tais resultados podem não ser totalmente aplicáveis aos países em desenvolvimento.

### Uma breve história do estudo ORCHESTRA

Para ajudar a preencher a lacuna referida, em 2014, o estudo ORCHESTRA (*ORganizational CHaractErSTics in cRitical cAre*) foi concebido para descrever a estrutura, o processo e a organização das UTIs, além de investigar o impacto dessas características nos desfechos dos pacientes e no desempenho e eficiência nos cuidados intensivos. O estudo foi desenhado em fases, para propor hipóteses consistentes com o conhecimento atual e incluir novos centros e pacientes em cada fase. No início, incluiu exclusivamente UTIs brasileiras, mas, em fases mais recentes, também foram incluídas UTIs do Uruguai. A figura 1 mostra o número de pacientes e centros incluídos em todas as fases. Nas três primeiras fases, foram incluídos mais de 475 mil pacientes em mais de 200 UTIs. No entanto, o estudo foi interrompido durante a pandemia da doença do coronavírus 2019 (COVID-19) porque as UTIs e as instalações hospitalares foram gravemente afetadas; além disso, também foram afetados os dados dos casos dos pacientes.^(3)^ Atualmente, a quarta fase está em andamento e inclui internações em UTIs de 2022 a 2023. O Apêndice 1S (Material Suplementar) apresenta a lista de centros e investigadores que participaram de todas as fases.

**Figura 1:** Número de hospitais, unidades de terapia intensiva e pacientes incluídos em cada uma das quatro fases do ORCHESTRA. O estudo não foi realizado entre 2019 e 2021. * A Fase 4 ainda está em andamento. O número final de hospitais, unidades de terapia intensiva e pacientes ainda não é conhecido. UTI - unidade de terapia intensiva.

### Desenho e metodologia do estudo

Uma descrição completa dos métodos é fornecida no Apêndice 2S (Material Suplementar). Resumidamente, o ORCHESTRA é um estudo de coorte retrospectivo multicêntrico que usou dados coletados prospectivamente de admissões consecutivas em UTIs. O estudo usa uma abordagem pragmática. Foram convidadas a participar as UTIs registradas na *Brazilian Research in Critical Care Network* (BRICNet)^(4)^ que utilizam o Epimed Monitor System (Epimed Solutions, Rio de Janeiro, Brasil),^(5)^ um sistema de registro comercial baseado em nuvem para fins de melhoria da qualidade e *benchmarking*. Os dados anonimizados de todos os pacientes adultos (≥ 16 anos de idade) são obtidos do Epimed Monitor System. Foram excluídos os pacientes readmitidos ou cujos dados principais (por exemplo: diagnóstico de admissão e desfechos hospitalares) estivessem faltando. Os dados são inseridos prospectivamente em um formulário eletrônico estruturado de relato de caso por meio de uma combinação de dados integrados com prontuários eletrônicos de saúde locais e dados inseridos manualmente por um gestor de caso treinado. Os dados coletados incluem dados demográficos, diagnósticos, comorbidades e avaliações de fragilidade; pontuações usadas regularmente em cuidados críticos, incluindo o *Simplified Acute Physiology Score* (SAPS) 3; uso de suporte de órgãos; e desfechos de UTI e hospitalares, entre outras variáveis. Todas as variáveis do Epimed Monitor System são estruturadas com códigos internos vinculados, sem campos de texto livre, com procedimentos e controles para auxiliar na entrada de dados e minimizar erros de processamento e o registro de valores discrepantes ou implausíveis.

Em seguida, o coordenador da UTI e/ou o enfermeiro-chefe preenchem um formulário *on-line* sobre as características organizacionais, estruturais e de processo do hospital e da UTI. Os domínios do inquérito baseiam-se na literatura em todas as fases do estudo e incluem, por exemplo, caracterizações da UTI e do hospital, padrões de pessoal, rodadas multidisciplinares, uso de listas de verificação, implementação de protocolos para evitar complicações associadas à assistência médica e a políticas de assistência à família. O desfecho primário do estudo é a mortalidade intra-hospitalar. Os desfechos secundários incluem mortalidade na UTI e as durações das internações na UTI e hospitalar. Além disso, são avaliadas medidas de desempenho e eficiência da UTI.^(6,7)^

Os dados anonimizados dos pacientes e das UTIs são processados e analisados de forma centralizada em servidores dedicados com controle de acessos e registros em conformidade com as normas de privacidade e proteção de dados.

### Principais publicações e resultados

Os resultados de todas as publicações relacionadas ao ORCHESTRA estão resumidos na tabela 1S do Material Suplementar; no entanto, há alguns estudos em andamento. Apresentamos aqui uma amostra selecionada dos principais resultados.

No primeiro estudo, o número de protocolos totalmente implementados e de protocolos clínicos de gestão conjunta (mais de um prestador de cuidados envolvido) foi associado à menor mortalidade.^(8)^ Em um estudo subsequente em pacientes com câncer, além do número de protocolos, a presença de farmacêuticos dedicados na UTI e a ocorrência de reuniões diárias entre oncologistas e intensivistas no planejamento de cuidados foram associadas a taxas de mortalidade mais baixas e ao uso mais eficiente de recursos.^(9)^ *Checklists* e protocolos também são essenciais para garantir a continuidade da qualidade dos cuidados durante os fins de semana, especialmente em pacientes com cirurgia programada.^(10)^ A implementação de protocolos associada a melhores desfechos é um achado recorrente de vários estudos relacionados ao ORCHESTRA (Tabela 1S - Material Suplementar) e contrasta com os achados de estudos realizados em países desenvolvidos.^(11,12)^ Podemos levantar a hipótese de que, em um cenário com menor concentração de enfermeiros e outros prestadores de cuidados por paciente, os processos protocolizados para prevenir complicações associadas à assistência médica e aderir às melhores práticas de evidência contribuem para atenuar os efeitos na continuidade do atendimento. A análise de agrupamentos com aprendizado de máquina foi usada para investigar se os padrões relacionados à equipe estavam associados a melhores desfechos.^(13)^ As unidades de terapia intensiva pertencentes ao agrupamento com intensivistas em tempo integral, farmacêuticos dedicados e níveis mais altos de autonomia do enfermeiro tiveram os melhores desfechos.

Além disso, aproveitamos a oportunidade para avaliar e validar vários escores usados rotineiramente em cuidados intensivos. Por exemplo, o SAPS 3, recomendado pela Associação de Medicina Intensiva Brasileira (AMIB) para avaliar o desempenho da UTI no Brasil, só foi validado em estudos com um número limitado de instituições e pacientes. Um estudo de validação usando o banco de dados ORCHESTRA revelou que a equação padrão do SAPS 3 foi bem ajustada para recomendação no Brasil. Notavelmente, também validamos a razão de uso de recursos padronizada como uma medida de eficiência no uso de recursos na UTI.^(8,14)^

## CONCLUSÃO

O estudo ORCHESTRA é um dos maiores estudos contemporâneos de coorte em todo o mundo e contribuiu para identificar alvos potencialmente modificáveis para melhorar a organização da UTI e a assistência ao paciente. Além disso, foi interessante validar instrumentos relevantes para caracterizar e estratificar pacientes em estado crítico e avaliar o desempenho e a eficiência da UTI. As perspectivas futuras para as próximas fases incluem UTIs de outros países onde seja possível atender aos critérios de elegibilidade do estudo.
